# The creation of synthetic crystalline bovine insulin

**DOI:** 10.1007/s13238-015-0221-x

**Published:** 2015-10-20

**Authors:** Yeping Sun

**Affiliations:** CAS Key Laboratory of Pathogenic Microbiology and Immunology, Institute of Microbiology, Chinese Academy of Sciences, Beijing, 100101 China

**Figure 1:**
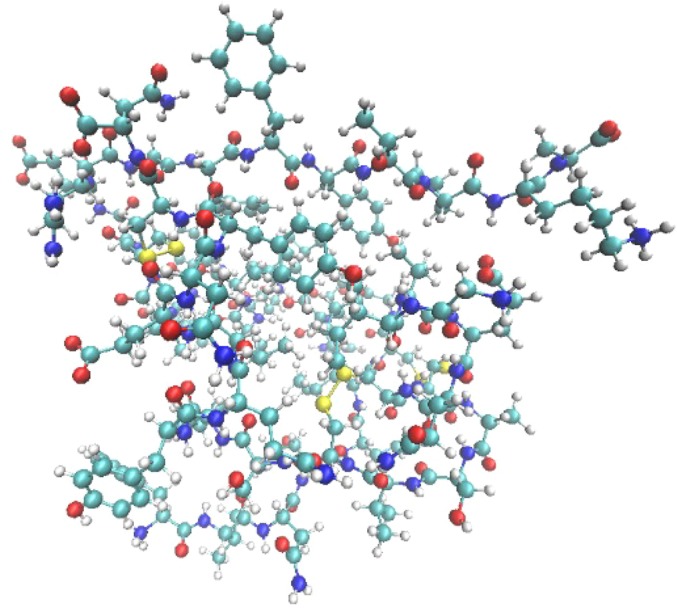
The structural model of bovine insulin. C, N, O, S and H atoms are indicated as cyan, blue, red, yellow, and white, respectively

Fifty years ago, a great achievement in life science occurred in China—the complete synthesis of crystalline bovine insulin—which gave Chinese scientists a sense of great elation and pride. Insulin (Fig. 1) is a hormone secreted by β cells in pancreas. Before the clinical application of insulin, diabetes was a feared disease that commonly led to death. Insulin has been studied since 1868 when Paul Langerhans, a medical student in Berlin found clusters of cells in the pancreas (Langerhans, 1868). These were later called ‘‘Islets of Langerhans’’. Some of these cells were eventually shown to produce insulin. The term ‘‘insulin’’ origins from ‘‘Insel’’, the German word for “islet” or “small island” (Sakula, 1988). Frederick Grant Banting, a young Canadian physician first extracted insulin from the pancreas of a dog whose pancreatic duct had been surgically ligated at University of Toronto in 1921, with the experimental facilities provided by Prof. John James Rickard Macleod and the assistance of one of Macleod’s students, Charles H. Best. Biochemist James Bertram Collip helped purify the extract. For this work Banting and Macleod shared the 1923 Nobel Prize in Physiology or Medicine (Nobelprize.org, 2014).

**Figure 2:**
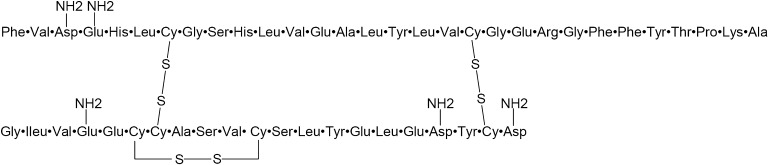
The primary structure of insulin. Adapted from *Science* 129, 1340–1344 (1959)

British molecular biologist Frederick Sanger determined the primary structure of insulin through 10 years of research: it comprises of two chains, chain A and chain B; chain A contains 21 amino acid residues while chain B consists of 30 residues; the two chains are linked by two disulfide bonds and there is an intra-chain disulfide bond in chain A (Fig. 2) (Sanger, 1959). This is the first protein structure determined in human history, which Sanger was awarded the 1958 Nobel Prize in Chemistry for.

Driven by the “great leap forward” campaign in 1958, Shanghai Institute of Biochemistry, Chinese Academy of Sciences and Perking University proposed that China should artificially synthesize insulin and obtained the support of the Chinese government. The project started in 1959, however, at that time, there was a lack of adequate equipment, the raw materials of amino acids and other necessary reagents. Consequently, synthesis of such a large compound represented a formidable task. The strategy adopted was to involve as many capable scientists as possible with eventually several hundreds of participants from eight different institutes participating in the project. People worked day and night preparing amino acids and other reagents, purifying solvents and synthesizing small peptides.

In 1963, Panayotis G. Katsoyannis (Katsoyannis et al., 1963) at Pittsburgh in U.S.A and Helmut Zahn (Zahn and Schade, 1963) at Aachen in Germany reported that they had synthesized insulin with weak activity. These reports placed a great deal of pressure on Chinese researchers and gave them further motivation to complete their project and be first to synthesize insulin with full activity. In order to accomplish this task three teams were chosen to work together synergistically in Shanghai: Prof. Qiyi Xing of Perking University was to lead his group to Shanghai and combine with Prof. You Wang’s group in Shanghai Institute of Organic Chemistry, Chinese Academy of Sciences where they were to be responsible for the synthesis of chain A; Prof. Jingyi Niu of Shanghai Institute of Biochemistry, Chinese Academy of Sciences and his team would complete the synthesis of chain B; and Prof. Chenglu Zhou’s team in Shanghai Institute of Biochemistry, Chinese Academy of Sciences would be responsible for the split and recombination of chain A and B.

**Figure 3:**
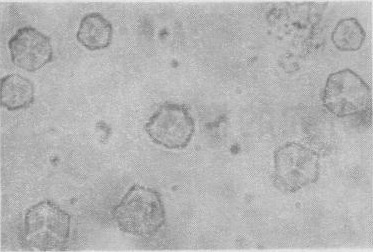
The crystalline bovine insulin. Adapted from *Scientia Sinica* 14, 1710–1716 (1965)

In August of 1964, Prof. Jingyi Niu successfully synthesized chain B and assembled it with chain A of natural insulin into semi-synthesized bovine insulin (Niu et al., 1964). In May of 1965, the combined team of Perking University and Shanghai Institute of Organic Chemistry finished the synthesis of Chain A of insulin (Wang et al., 1966). At the same time, Prof. Chenglu Zhou’s team greatly increased the yield of the recombination of chain A and B (Institute of Biochemistry, Chinese Academy of Sciences, 1966). And finally, the fully synthetic bovine insulin came into production on September 17, 1965. This is the first totally synthetic insulin in crystallized form (Fig. 3) with full biological activity, immunogenicity and chemical property in the world.

This work was published in *Scientia Sinica* (Kung et al., 1965) which aroused a great deal of international interest. *Science* magazine reported this achievement in July, 1966 (McElheny, 1966). Prof. Henry Norman Rydon, a celebrated statesman in peptide chemistry reviewed in *New Scientist* magazine the reasons why the competitors of the Chinese scientists had failed to obtain insulin with full activity and commented that the achievement of the Chinese scientists was “a truly seminal piece of work which would stimulate and encourage work directed towards the synthesis of larger, more typical, proteins” (Rydon, 1966).

The synthesis of a complete and active insulin in only six years was a fantastic achievement given that it was predicted to take much longer. Why was such a brilliant and awesome achievement first accomplished in China, a developing country where the basis of scientific research was relatively weak, rather than in developed countries such as America and Germany? Besides the timely decision-making and strategic planning of the scientific administrative department in the government, the most important determinants might be the laudable mentality of Chinese scientists at that time, which can be summed up into “insulin spirit”, which includes four aspects: (1) selfless dedication. All people involved in the project devoted all themselves into the demands of the project without considering their own interest; (2) honesty. Every intermediate in more than 200 steps of the synthetic procedure had to be rigorously identified, so even a slight problem on these identification procedures might lead to total failure of the whole project; (3) close cooperation. The three teams organized from three different institutes had clear assignment of their responsibilities and worked synergistically for the common aim of the project, so that high efficiency was achieved; (4) the spirit of welcoming challenges. To synthesize a protein consisting of 51 amino acids was a formidable task, so it was the spirit of welcoming a challenge that helped the Chinese scientists gain the respect of the world. Today, the “insulin spirit” is still of great value in constructing our research systems and managing research projects. Most importantly, it has become the source of confidence and strength of every Chinese researcher.
